# The impact of herd age structure on the performance of commercial sow-breeding farms

**DOI:** 10.1186/s40813-024-00406-5

**Published:** 2024-11-23

**Authors:** Santos Sanz-Fernández, Cipriano Díaz-Gaona, João Simões, José Carlos Casas-Rosal, Nuria Alòs, Llibertat Tusell, Raquel Quintanilla, Vicente Rodríguez-Estévez

**Affiliations:** 1 Departamento de Producción Animal, IC Zoonosis y Enfermedades Emergentes ENZOEM, Facultad de Veterinaria, Campus de Excelencia Internacional Agroalimentario, Universidad de Córdoba, Campus de Rabanales, Córdoba, 14071 España; 2 Universidade de Trás-os-Montes e Alto Douro, Vila Real, Portugal; 3 Departamento de Matemáticas, Universidad de Córdoba, Avd. San Alberto Magno s/n, Córdoba, 14071 España; 4 Animal Breeding and Genetics Program, Institute of Agriculture and Food Research and Technology (IRTA), Caldes de Montbui, 08140 Spain

**Keywords:** Replacement rate, Census structure, Parity, Breeding sows, Reproductive performance

## Abstract

**Background:**

The herd age structure, i.e., distribution of sows within a farm based on their parity number, and its management are essential to optimizing farm reproductive efficiency. The objective of this study is to define different types of herd age structure using data from 623 Spanish commercial sow farms. Additionally, this study aims to determine which type of herd age structure can enhance reproductive efficiency at the farm level.

**Results:**

Farms are classified into three groups according to the quadratic function fitted to the percentage of sows by parities. This classification unveils three types of herd structures: type 1 (HS1) exhibits a concave-downward trend, with a higher percentage of sows in intermediate parities (mean of 45.5% sows between the 3rd to 5th parity); type 2 (HS2) presents a trend curve that is close to a straight line, with a gradual decrease in the percentage of sows per parity (approximately 2% loss of sows census per parity); and type 3 (HS3) shows an upward concave trend curve, with an increase in the percentage of sows in later parities (19.0% of sows between 7th and ≥ 8th parity). Parametric tests assess productivity differences between the three types of herd structures (*p* < 0.01). HS1 farms have the best productive outcomes over a year, with 31.2 piglets weaned per sow and year (PWSY) and a farrowing rate of 87%, surpassing HS2 and HS3 farms (30.1 and 28.7 PWSY; 85.3% and 83.4% farrowing rates, respectively). HS1 also have the lowest percentage of sows returning to oestrus (11.8%) and the highest number of weaned piglets per litter (12.8), compared to HS2 (13.2% and 12.4 piglets weaned) and HS3 (15.1%, 11.9 piglets weaned). These differences show a medium effect size (η^2^ between 0.06 to < 0.14).

**Conclusions:**

This study shows the importance of herd age structure on sow-breeding farms as a factor of reproductive efficiency. The results endorse the proposed classification based on the curvature of the trend parabola obtained with the quadratic function to categorize herd structures into three groups. Additionally, these findings highlight the importance of considering the herd age structure in farm decision-making.

## Background

One crucial aspect for achieving optimal farm efficiency is to control the herd age structure of breeding sows [1, 2], because it directly influences the number of piglets produced within a herd [3]. The herd age structure refers to the distribution of sows within a farm based on the number of reproductive cycles or parities. In general, the herd age structure can be divided into three main groups of sows [4]: gilts and primiparous sows, which exhibit lower prolificacy and fewer weaned piglets; mid-parity sows (third to fifth parity), with the highest productivity; and older sows, encompassing sows from the sixth to eighth parity or older, which are those close to being culled and have physiological traits that decrease their productivity. In addition, piglet survival rates also vary across sow reproductive cycles [5] due to variations in colostrum and milk production, immunoglobulin concentration, and birth weight within litters [6–8]. Therefore, to ensure optimal farm performance, it is crucial to study the distribution of sows across parities (herd age structure), as well as to consider other reproductive parameters such as the number of piglets weaned per sow and year (PWSY), the number of farrowings per sow per year, and sow lifetime performance [9].

Thus, the proportion of sows within each parity group, determined by sow removal and by the maximum number of productive parities at which sows are culled, is a crucial factor in the functionality, productivity and profitability of a pig farm. Carroll [10] defined the ideal herd age structure as one that has a gradually decreasing percentage of sows from 1st to 8th parity, which has been widely accepted as the best approach for ensuring farm efficiency. This structure suggests a 1st parity sow percentage of ≥ 17%, around 42% of sows in the 3rd to 5th parity, and a maximum 8th parity sow percentage of ≤ 4%. More recently, several authors have also recommended maintaining the percentage of 1st parity sows between 15 and 20% of breeding sows [1, 3, 11–13]. In this same line, Koketsu [4] recommended maintaining a stable census, with stable subpopulations of mid-parity sows and mated gilts to optimise herd productivity. Nevertheless, many farms have an unstructured sow herd distribution derived from challenges in sow culling or replacement programs, which results in worse productivity parameters.

Despite this evidence, the impact of herd age structure on farm productivity has barely been analysed. The objective of this study is to define different types of herd age structures by studying 623 Spanish commercial sow farms in order to assess the relationship between herd age structure and farm performance, and to determine which type of herd age structure can enhance pig farm reproductive efficiency.

## Materials and methods

### Data source

The dataset analysed in the present study comes from the BDporc^®^ databank [14] within the framework of a collaboration agreement between the Institute of Agrifood Research and Technology (IRTA) and the Department of Animal Production at the University of Cordoba. The BDporc^®^ is the main database of sow-breeding farms in Spain, where performance characteristics are collected, through the periodic submission of data generated on the farms, gathered in their own software for data collection and management. The analysed dataset was collected in 2020 from 623 intensive, indoor commercial farms, with a census of 888,479 reproductive sows, representing approximately 40% of the sow-breeding census in Spain. These farms use partially slatted floors, with a maximum gestation period of 4 weeks in stalls, in compliance with current animal welfare legislation [15].

For confidentiality reasons, there is no information available on the genetics of the sows from the studied farms. Nevertheless, these sows are derived from modern commercial lines, primarily hybrids of Landrace and Large White crosses. Farms with Iberian breed sows were not included, as the BDPorc database clearly distinguishes these Iberian breed farms from the rest. Therefore, the results of this study can be extrapolated beyond Spain, the largest pork producer in Europe and the third globally [16].

Regarding the farms included in the study, they should have records up to at least 6th parity in order to evaluate their census structure. A total of 8 farms were excluded from the study for not having data up to 6th parity. Since most of the sows do not reach 8 or more parities and for the sake of simplification, BDporc groups sows with 8 or more parities into a single group. Similarly, these oldest parities are grouped in other studies of intensive farms [4] because those sows are close to be culled [17, 18].

The productive parameters analysed at the farm level correspond to the data collected over one year, from January to December 2020, and are annual farm averages: number of sows on the farm; replacement rate (proportion of sows newly introduced into the farm relative to the average number of sows present); piglets weaned per sow per year (PWSY); age of sows at culling (months); farrowings per culled sow (average of total farrowings performed by sows until their culling); total number of piglets weaned per culled sow (total number of piglets produced by a sow throughout her life, until its culling); farrowings per sow and year; farrowing rate; percentage of sow return to oestrus; weaning-to-first-service interval (WSI); weaning-to-oestrus interval (WOI); weaning to conception interval (WCI); number of total born (TB), born alive (BA), stillborn (SB) and weaned (W) piglets per litter; mortality rate of TB piglets at weaning; and mortality rate of BA piglets at weaning.

To analyse the census structure, the percentage of sows within each parity has been calculated, using the total number of litters recorded for each parity over a year (*N* = 1,860,663 litters). Additionally, the average number of BA and W piglets as well as WCI are analysed by parity.

### Modelling herd age structure and classification of farms into groups

The farms have been classified into three groups based on the quadratic function of the herd age structure of each farm, where the dependent variable is the percentage of sows, and the independent variable is the parity of the sow (1st to ≥ 8th parity). This function was selected because it is easy to interpret and provides a good fit for the real distribution of sow census across parities within farms. Additionally, it enables the assessment of non-linear relationships between the parity number and the sow census, offering a high degree of flexibility to accurately capture complex patterns and variations in the data. To compare the goodness of fit between the different functional forms, the Akaike information criterion has been used. The graphical representation of a quadratic function is a parabola, defined by the equation: f(x) = ax^2^ + bx + c. The coefficients of the quadratic regression provide information about the shape of the curve and the relationship between variables. Thus, the coefficient “a” determines the orientation of the graph, indicating the curvature of the function and whether the parabola opens upwards or downwards [19]. Moreover, the absolute value of “a” determines the magnitude of the parabola’s curvature and its direction. This “a” coefficient was calculated using the least squares method for quadratic functions using the editor of Python V.3.10.10, Pyzo V.4.12.8.

Accordingly, the three groups of herd age structure have been defined based on the value of the coefficient “a”, classifying the farms according to the 25th, 50th, and 75th percentiles (extreme values and median) of this coefficient; these three groups of herd age structures are: type 1 (HS1), corresponding to the percentile 25, with the lowest value of the coefficient “a” (negative values; *N* = 156 farms); type 2 (HS2), corresponding to the percentile 50 of the coefficient “a” (values closest to zero; *N* = 311 farms); type 3 (HS3), corresponding to the percentile 75, with the highest value of the coefficient “a” (positive values; *N* = 156 farms). For each group of herd age structure, its quadratic function has been calculated, obtaining the coefficient of determination (R^2^) and the Root Mean Square Error (RMSE). Additionally, the linear function of each herd age structure group has been calculated in parallel to compare and validate the fit of the herd age structure to a quadratic model.

### Statistical analysis

Statistical analyses of the dataset were performed using IBM SPSS^®^ 22 software. Descriptive statistics were calculated for the productive parameters of all farms and for each parity, as well as for sow distribution.

To compare the three types of herd age structure, parametric tests were conducted after assessing normality using skewness and kurtosis calculations; to meet normality criteria established by Kline [20], skewness and kurtosis values were required to range from − 3 to 3 and between − 8 and 8, respectively. Specifically, the ANOVA test was performed, followed by the Tukey HSD test to analyse the differences in distribution between the three types of herd age structure compared to their productive outcomes. Furthermore, for cases where significant differences were observed, the effect size (η^2^) was calculated to measure the magnitude of the differences found. The effect size of an ANOVA is the value that measures how much the independent variable or factor (the type of herd age structure) influences the dependent variable (the productive parameters). Cohen [21] provides classification benchmarks for effect size levels, defining small effects (η^2^ = 0.01 to < 0.06), medium effects (η^2^ = 0.06 to < 0.14), and large effects (η^2^ ≥ 0.14).

## Results

### Evaluation of the productive parameters of the farms

Descriptive statistics of the productive variables associated with the performance of the studied farms are shown in Table 1. In general, the census of the studied farms exceeded 1400 breeding sows, with a mean annual productivity of 29.73 PWSY and a mean of 2.43 farrowings per sow per year.

**Table 1:** Descriptive statistics for the performance characteristics of the commercial farms included in the study (*N* = 623)

	Mean	Standard deviation	Percentiles		
			25	50	75
Mean number of sows on the farm	1426.13	1217.06	561.64	983.11	2057.64
Replacement rate (%)	47.31	13.10	40.19	45.78	52.76
Number of piglets weaned per sow per year	29.73	3.44	27.47	29.47	32.04
Culled sow age (months)	32.75	4.61	30.10	32.62	35.11
Farrowings per culled sow	4.55	0.90	4.05	4.57	5.06
Total number of piglets weaned per culled sows	54.73	11.99	47.66	54.80	61.50
Farrowings per sow and year	2.43	0.08	2.40	2.44	2.48
Farrowing rate (%)	84.62	5.74	81.54	85.34	88.29
Percentage of sows return to oestrus	13.95	5.58	10.27	13.02	17.13
Weaning-to-first-service interval (WSI, days)	6.17	1.75	5.20	5.72	6.58
Weaning-to-oestrus interval (WOI, days)	4.90	0.73	4.49	4.80	5.15
Weaning to conception interval (WCI, days)	9.37	3.40	7.25	8.65	10.48
Number of piglets total born (TB, per litter)	15.63	2.01	14.16	15.08	17.33
Number of piglets born alive (BA, per litter)	14.30	1.71	13.06	13.89	15.79
Number of piglets still born per litter	1.33	0.48	1.02	1.27	1.59
Number of piglets weaned per litter	12.22	1.36	11.21	12.06	13.15
Mortality rate of BA piglets at weaning (%)	14.41	4.53	11.57	14.08	17.33
Mortality rate of TB piglets at weaning (%)	18.41	6.97	12.58	19.10	23.55

In terms of sow longevity, the average age of culled sows was approximately 33 months, with a mean of 4.55 farrowings and 54.73 piglets weaned per sow lifetime. These farms had a mean replacement rate of 47.31%, with a mean percentage of sows returning to oestrus of nearly 14%, and a mean WCI of 9.37 days.

**Fig. 1:**
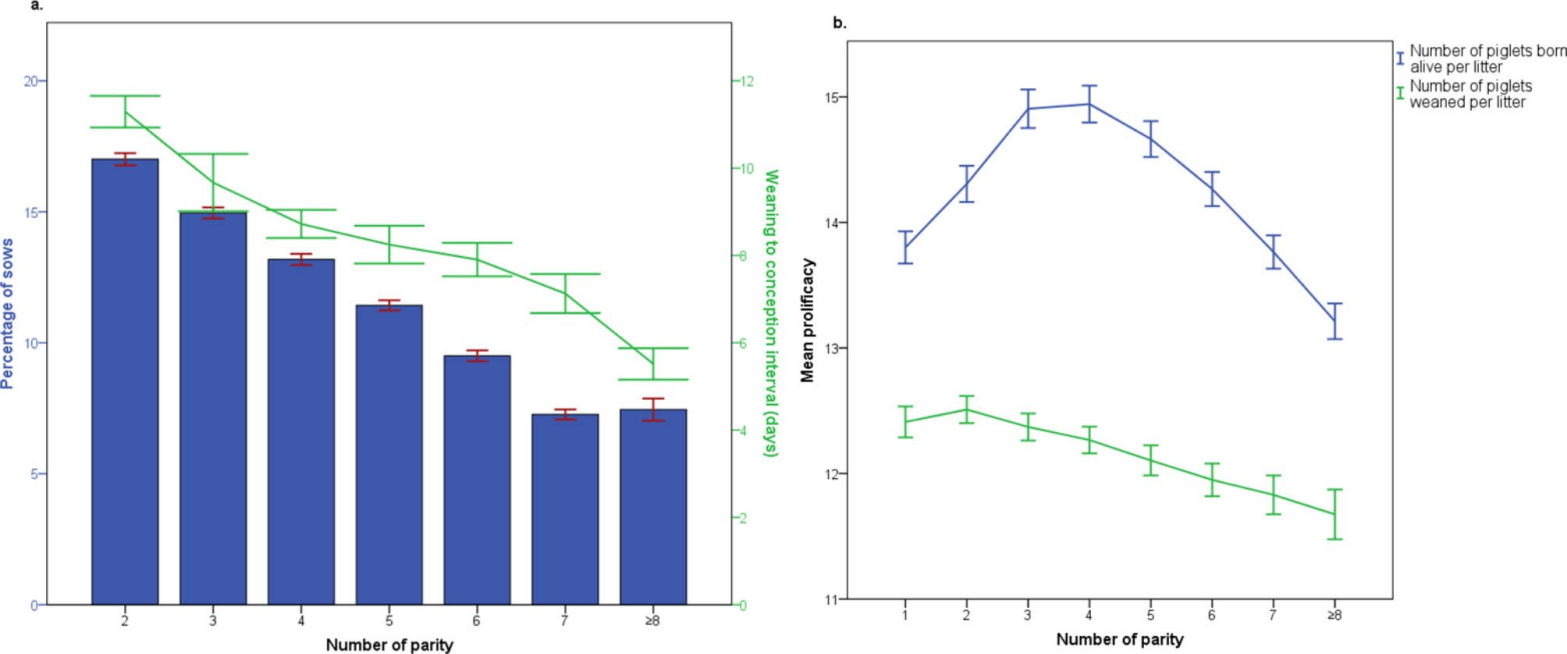
Descriptive statistics for **(a)** the sow distribution and weaning to conception interval per parity; and **(b)** prolificacy for piglets born alive and weaned per parity (*N* = 623) Bars (I) represent standard error of the mean (SEM)

Figure 1 shows the descriptive statistics for sow distribution, prolificacy and WCI per parity for the total number of farms. Thus, the herd age structure of all farms shows a gradual decrease in the percentage of sows from the 1st parity (with a mean of 19.58% sows) to the 7th parity (with a mean of 7.18% sows), approximately maintaining this percentage in the ≥ 8th parities.

Regarding litter size, the highest prolificacy for BA piglets was achieved at the 3rd and 4th parities, with means of 14.91 and 14.94 piglets, respectively, while the 2nd parity had the highest number of W piglets (12.51 piglets). On the other hand, the WCI gradually decreased as the number of parities increased, with a mean difference of nearly 6 days between the 2nd and ≥ 8th parities.

### Types of herd age structure

**Fig. 2:**
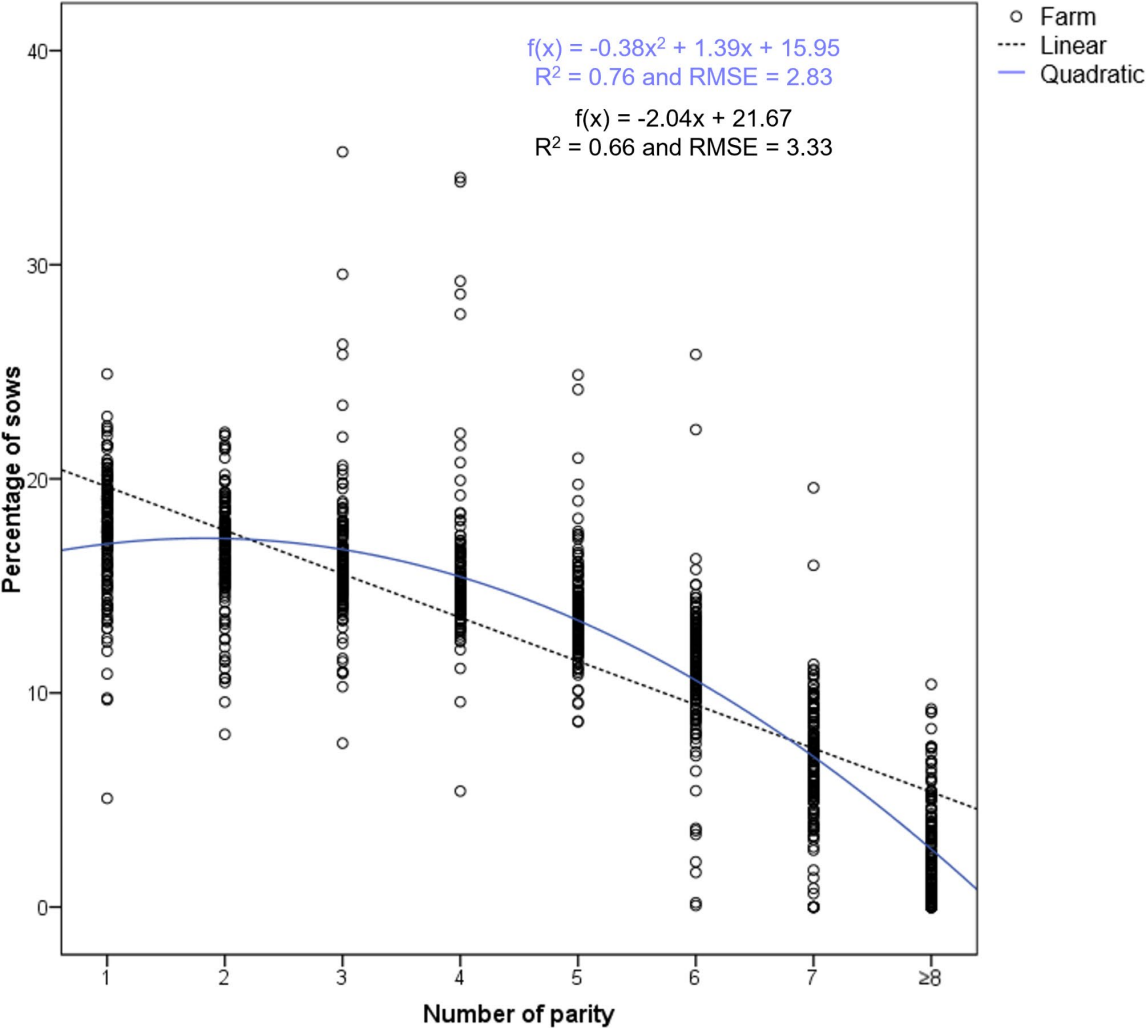
Quadratic function representation for Herd Structure Type 1 (*N* = 156). The mean and median of each cluster of data points at each parity can be found in Table 2

**Fig. 3:**
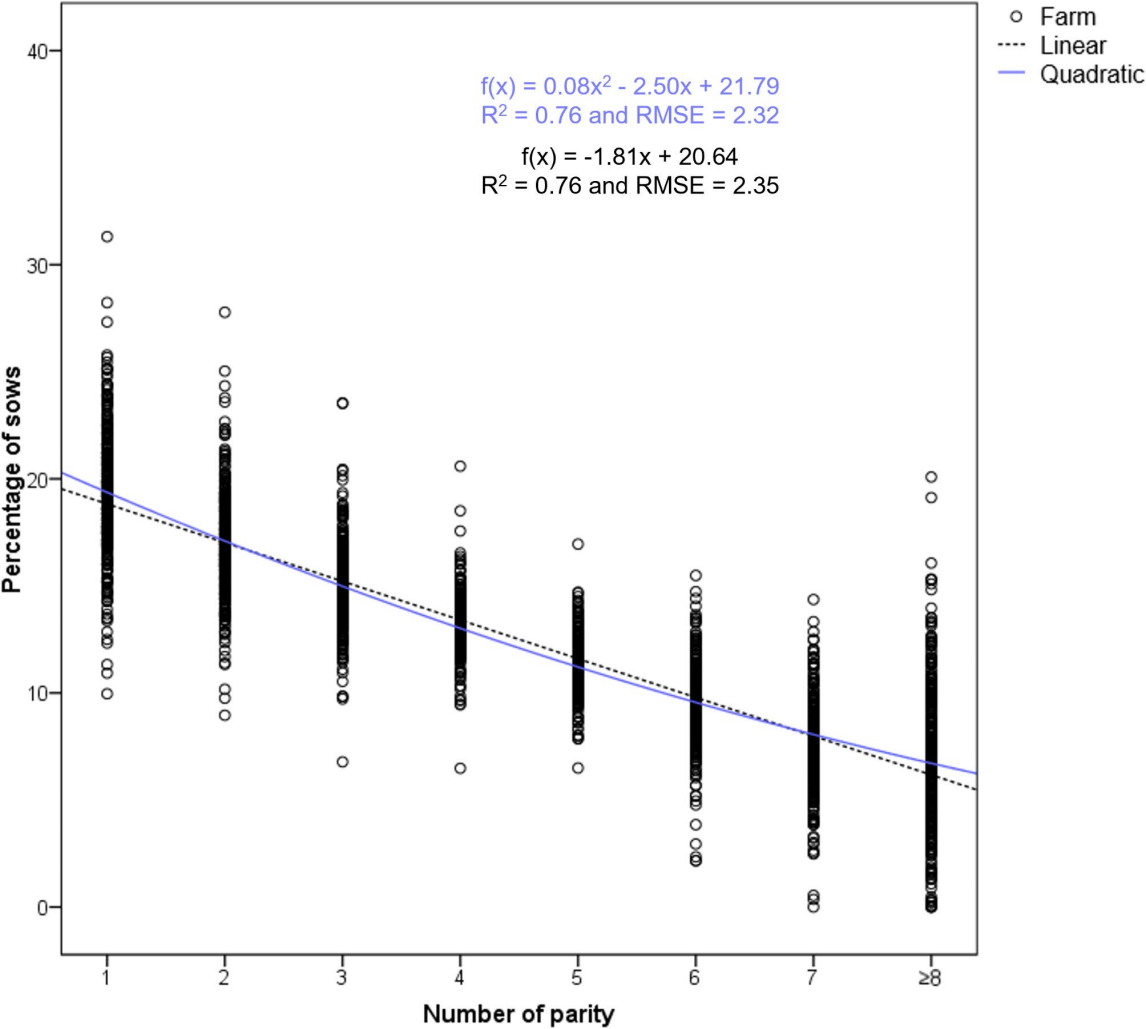
Quadratic function representation for Herd Structure Type 2 (*N* = 311). The mean and median of each cluster of data points at each parity can be found in Table 2

**Fig. 4:**
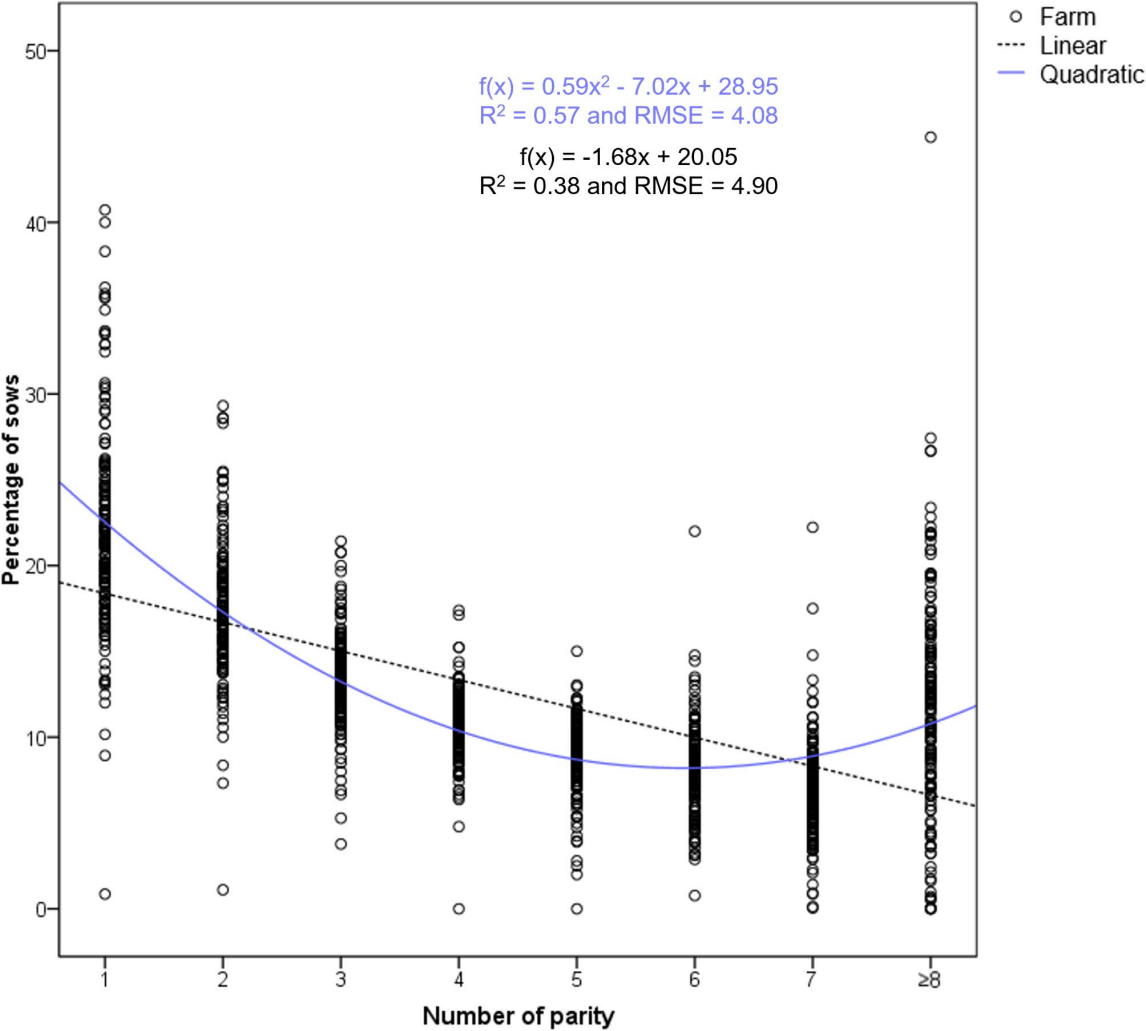
Quadratic function representation for Herd Structure Type 3 (*N* = 156). The mean and median of each cluster of data points at each parity can be found in Table 2

The farms were classified into three groups of herd age structure (HS1, HS2 and HS3); as stated above this was determined by the coefficient “a” of the quadratic function fitted to the distribution of sows by parity (Figs. 2, 3 and 4). Table 2 shows the percentage of sows at each parity (mean and median), the prolificacy (TB and BA) and W piglets per parity for these three groups of farms.

**Table 2:** Descriptive statistics for the sow distribution and prolificacy for piglets born alive and weaned at each parity according to the groups of herd age structures (*N* = 623)

		Parity 1	Parity 2	Parity 3	Parity 4	Parity 5	Parity 6	Parity 7	Parity ≥ 8
Herd Structure Type 1 (a25%) (*N* = 156)	Percentage of sows (mean)	17.55	16.57	16.25	15.44	13.85	10.99	6.80	2.56
	Accumulative percentage of sows	-	34.12	50.36	65.80	79.65	90.64	97.44	100
	Percentage of sows (median)	17.73	16.78	15.93	14.74	13.60	11.12	7.16	1.98
	Number of piglets total born	15.30	15.65	16.53	16.70	16.56	16.31	15.43	14.06
	Number of piglets born alive	14.24	14.69	15.35	15.31	15.00	14.59	14.12	13.71
	Number of piglets weaned	12.94	13.06	12.90	12.79	12.57	12.41	12.37	12.32
Herd Structure Type 2 (a50%) (*N* = 311)	Percentage of sows (mean)	19.30	17.03	15.06	13.21	11.37	9.45	7.47	7.11
	Accumulative percentage of sows	-	36.33	51.39	64.61	75.97	85.42	92.89	100
	Percentage of sows (median)	19.22	17.00	15.03	13.21	11.37	9.45	7.47	7.11
	Number of piglets total born	14.80	15.18	15.96	16.20	16.10	15.85	15.43	14.87
	Number of piglets born alive	13.70	14.21	14.81	14.85	14.59	14.18	13.69	13.16
	Number of piglets weaned	12.34	12.40	12.28	12.14	11.98	11.79	11.63	11.53
Herd Structure Type 3 (a75%) (*N* = 156)	Percentage of sows (mean)	22.17	17.42	13.45	10.78	9.05	8.12	7.00	12.01
	Accumulative percentage of sows	-	39.58	53.04	63.82	72.87	80.99	87.99	100
	Percentage of sows (median)	21.40	16.91	13.43	10.95	9.34	8.15	6.87	12.13
	Number of piglets total born	14.66	15.13	15.87	16.03	15.91	15.81	15.39	14.46
	Number of piglets born alive	13.57	14.12	14.66	14.76	14.47	14.11	13.57	12.86
	Number of piglets weaned	12.01	12.17	12.03	11.99	11.88	11.81	11.70	11.37

HS1 is characterised by maintaining a higher percentage of sows in the intermediate parities (an average of 45.5% sows between the 3rd to 5th parity; Table 2). The obtained quadratic function for this group shows a concave-downward trend curve (Fig. 2). HS1 farms had a negative coefficient “a” (ranging from − 1.8509 to -0.1346). The quadratic function representing the herd age structure is as follows: f(x) = -0.38 × ^2^ + 1.39x + 15.95, with a coefficient of determination R^2^ = 0.76 and RMSE = 2.83 (*p* < 0.001). The coefficient of determination R^2^ indicates that approximately 76% of the variation in the herd age structure within this group of farms can be explained by the quadratic function, and the predicted values by the quadratic function differ from the actual values by approximately 2.83 units on average, indicating a good fit of the model.

HS2 is characterised by a trend curve that is close to a straight line, with a gradual decrease in the percentage of sows from 1st to 8th parity, resulting in a loss of approximately 2% of the sow census as the number of parities increases (Table 2). HS2 farms had a coefficient “a” close to zero (ranging from − 0.1337 to 0.2916). The quadratic function representing the herd age structure is as follows (Fig. 3): f(x) = 0.08 × ^2^ − 2.50x + 21.79, with an R^2^ value of 0.76 and RMSE = 2.32 (*p* < 0.001).

HS3 is characterised by an upward concave trend curve, with an increase in the percentage of sows in the later parities (an average of 19.0% sows between the 7th to ≥ 8th parity; Table 2), compared to the other defined herd structure types. HS3 farms had a positive coefficient “a” (ranging from 0.2917 to 1.4762). The quadratic function representing the herd age structure is as follows (Fig. 4): f(x) = 0.59 × ^2^ – 7.02x + 28.95, with R^2^ = 0.57 and RMSE = 4.08 (*p* < 0.001), indicating a slightly larger average difference between predicted and observed values when compared with the other two types.

On the other hand, linear functions were defined for the 3 types of herd age structure, fitted to the distribution of sows by parity, depicted in Figs. 2 and 3, and 4, along with the quadratic functions. The linear regression models yield R^2^ values of 0.66, 0.76, and 0.38, and RMSE values of 3.33, 2.35, and 4.90, respectively, for HS1 farms (f(x) = -2.04x + 21.67), HS2 farms (f(x) = -1.81x + 20.64), and HS3 farms (f(x) = -1.68x + 20.05), indicating a generally poorer fit of these models compared to the previously defined quadratic functions.

## Comparisons of productive parameters depending on the herd age structure

The mean productive parameters of farms grouped according to their parity order distribution are shown in Table 3, showing significant differences between the three types of herd age structure.

**Table 3:** Mean (SD) of productive parameters according to the groups of herd age structures (*N* = 623)

	Herd Structure Type 1	Herd Structure Type 2	Herd Structure Type 3	*p*-value^1^	Effect Size^2^
Mean number of sows on the farm	1319.59 (1109.78)	1462.03 (1252.78)	1461.10 (1248.30)	0.451	0.00
Replacement rate	44.50^a^ (10.45)	47.31^ab^ (12.36)	50.16^b^ (17.94)	0.00**	0.02**
Number of piglets weaned per sow per year	31.17^a^ (3.27)	29.53^b^ (3.29)	28.71^c^ (3.44)	0.00**	0.07**
Culled sow age (months)	31.74^a^ (10.45)	32.83^ab^ (10.45)	33.61^b^ (10.45)	0.00**	0.02**
Farrowings per culled sow	4.44 (0.73)	4.58 (0.79)	4.62 (1.22)	0.20	0.01
Total number of piglets weaned per culled sows	55.37 (10.81)	54.43 (10.89)	54.68 (14.91)	0.73	0.00
Farrowings per sow and year	2.44^a^ (0.06)	2.44^a^ (0.08)	2.41^b^ (0.09)	0.00**	0.03**
Farrowing rate	87.02^a^ (4.36)	84.68^b^ (5.72)	82.11^c^ (5.96)	0.00**	0.09**
Percentage of sows return to oestrus	11.78^a^ (4.09)	13.85^b^ (5.64)	16.29^c^ (5.86)	0.00**	0.08**
Weaning-to-first-service interval (WSI)	5.80^a^ (1.06)	6.15^a^ (1.61)	6.60^b^ (2.37)	0.00**	0.03**
Weaning-to-oestrus interval (WOI)	4.78^a^ (0.52)	4.86^a^ (0.67)	5.11^b^ (0.95)	0.00**	0.03**
Weaning to conception interval (WCI)	8.37^a^(2.24)	9.37^b^ (3.42)	10.38^c^ (4.04)	0.00**	0.04**
Number of piglets total born	16.11^a^ (1.85)	15.52^b^ (2.01)	15.35^b^ (2.10)	0.00**	0.02**
Number of piglets born alive	14.79^a^ (1.56)	14.21^b^ (1.71)	14.00^b^ (1.76)	0.00**	0.03**
Number of piglets still born	1.33 (0.46)	1.32 (0.48)	1.35 (0.50)	0.76	0.00
Number of piglets weaned	12.75^a^ (1.33)	12.10^b^ (1.32)	11.90^b^ (1.34)	0.00**	0.06**
Mortality rate of BA piglets at weaning	13.64 (4.39)	14.65 (4.63)	14.71 (4.55)	0.048*	0.01*
Mortality rate of TB piglets at weaning	17.98 (7.09)	18.54 (7.01)	18.59 (6.91)	0.67**	0.00

Abbreviations: ^a-c^ Values within a row with different superscripts indicate significant differences between groups. ^1^*p*-value: * *p* < 0.05; ** *p* < 0.01

^2^Effect size (Cohen’s d) classification levels (Cohen, 1988): small (d = 0.01 to < 0.06), medium (d = 0.06 to < 0.14) and large (d ≥ 0.14) effects

HS1 farms, characterised by a slightly concave-downward trend curve, due to a higher percentage of sows in intermediate parities, have the lowest mean age for culled sow and the lowest replacement rate. Both values are not significantly different from those obtained in HS2 farms but are significantly lower than the age for culled sow and replacement rate of HS3 farms. The three herd structure types show significant differences regarding annual productivity (*p* < 0.01); HS1 farms exhibit the highest mean annual productivity (31.2 PWSY), while HS3 ones, characterised by a higher percentage of sows in the later parities, have the lowest one (28.7 PWSY). Additionally, HS1 farms also have the highest farrowing rate (87.0%), the lowest percentage of sows returning to oestrus (11.8%) and the shortest WCI (8.4 days) (*p* < 0.01). Similarly, the highest prolificacy (for TB and BA) and number of W piglets were also observed on HS1 farms, with means of 16.1 TB, 14.8 BA and 12.8 W, with significant differences between the groups (*p* < 0.01).

HS1 and HS2 farms have fewer non-productive days, with means of 4.8 and 4.9 days for WOI, and 5.8 and 6.2 days for WSI, respectively (*p* < 0.01). As a result, these types of farms have a higher number of farrowings per sow per year than HS3 farms (*p* < 0.01), both with 2.44 farrowings per sow per year.

Finally, the effect size of herd age structure on these productive parameters has been calculated, revealing that the greatest effects are for farrowing rate, percentage of sows returning to oestrus, annual productivity (PWSY) and W piglets, with a medium effect size, with values of η^2^ = 0.09, 0.08, 0.07 and 0.06, respectively. The remaining productive parameters showing significant differences among the different types of herd structure showed a small effect, with values of η^2^ < 0.6.

## Discussion

The present work addresses a study about herd age structure and other related reproductive parameters from data gathered in the Spanish Pig Database BDporc^®^.

The mean annual productivity of 29.73 PWSY indicates efficient breeding practices, aligning well with established industry standards in Spain [22]; therefore, these farms are considered to have high annual productivity [23]. While these farms demonstrate good productivity outcomes, it is essential to note that they show means of 1.33 SB piglets and pre-weaning mortality rates of 18.4% and 14.4% for piglets TB and BA, respectively, that should be improved. In this regard, these results show piglet survival rates below the minimum target suggested by Sanz-Fernández et al. [5] which are 83.2% and 88.5% for piglets TB and BA, respectively. Therefore, while these farms exhibit high productivity there is room for improvement.

In terms of prolificacy (BA) per parity, it is well known that this varies throughout the reproductive cycles of sows [24]. In this study, the 3rd to 5th parities prove to be the most productive cycles, in line with the parity curve pattern of litter size reported by Sell-Kubiak et al. [25]. A drop of almost two piglets was observed between these highly productive cycles and the one with the lowest prolificacy (parities ≥ 8). Besides that, as in the study by Lavery et al. [26], the WCI decreases as the number of parities increases, with a decline in the number of W piglets from the 3rd parity onwards.

Sow longevity, farrowings per culled sow, replacement rate and herd distribution are all inter-related measures. This study reports similar results to those found in a study of 110 commercial breeding herds in Japan by Koketsu [27], with a mean of 4.55 farrowings at culling and a 47.3% replacement rate. Although currently a replacement rate of 40–50% is considered appropriate for maintaining a proper herd age structure [28], keeping a sow on the farm for a longer time allows for a greater opportunity to recoup the initial investment [29]. According to Małopolska [30], the primary reasons for culling sows are reproductive problems, leading to an increase in replacement of sows. This, in turn, results in higher production costs and decreased profitability [18].

Furthermore, herd longevity is a concern not only from an economic and productive perspective but also from a consumer perspective of animal welfare. Hoge and Bates [31] suggest improving sow management to extend the productive life of breeding sows to improve both profitability and animal welfare. This is particularly relevant given the increasing societal awareness and concerns about animal welfare and sustainability [32]. Thus, the use of indicators such as herd longevity may be crucial for evaluating sustainability, animal welfare and management of breeding sows.

While several studies have examined models for sow herd management [33] and sow removal and culling patterns [1, 18, 34, 35], there remains a gap in understanding the impact of herd age structure on farm efficiency. This study bridges that gap by classifying farms into three distinct types or models of herd age structure based on the coefficient “a” of quadratic functions fitted to the percentage of sows per parity. Although this classification may seem simplistic and might not fully capture the complexity of herd age structure, its simplicity offers clear advantages to producers and technicians for benchmarking purposes, as it is easy to interpret and apply. Consequently, these models exhibit distinct shapes and characteristics, significantly contributing to our understanding of sow distribution patterns. Moreover, when analysing the influence of these three types of herd age structure on farms’ reproductive efficiency within a year, significant differences are observed. However, it is worth noting that, alongside the proposed methodology, exploring alternative methods for classifying farms based on their herd structure could be beneficial. This may include employing multivariate analysis techniques or simulation models to estimate the impact of various sow replacement and culling strategies on herd structure and farm productivity over time, as discussed by Plà et al. [36].

As a results, HS1 shows a downward concave trend, with a higher percentage of sows in the intermediate parities (3rd to 5th ). This distribution ensures that more than 90% of the sows on the farm are within the 1st to 6th farrowing, allowing them to reach their maximum reproductive potential, as these sows are the most productive [4]. Thereby, farms HS1 achieve the best productive outcomes, including a higher number of PWSY, surpassing the results of farms with other type of herd age structure.

Furthermore, Buxadé Carbó et al. [37] suggested maintaining a higher percentage of sows until the 3rd or 4th parity to maximise their depreciation. This implies that the percentage difference between the 1st and 2nd, and 2nd and 3rd cycles should be minimal, resembling the HS1 defined in this study, which has a higher percentage of sows in intermediate parities. This allows for the maximisation of the number of sows in the most productive parities, achieving higher productivity and reducing the average cost per piglet.

HS2 exhibits a trend curve closer to a straight line, maintaining a steady decline in the percentage of sows, aligning with the ideal herd age structure defined by Carroll [10]. This strategy aims to mitigate productivity variations attributed to unstructured herd distribution. On average, this group showed 19.3% of first-parity sows, slightly above the 17% indicated by Carroll [10] and below the 24.3% reported in the recent cohorts study by Bergman et al. [32]. Furthermore, the HS2 also aligns with the recommendations of Houška [1], who suggested that the percentage of sows from the 1st and 2nd parity should be similar to the percentage of sows from the 3rd to the 5th parity; which, in this study, represent 36.3% and 39.6% of the breeding sows census, respectively. Therefore, this census distribution is considered a highly stable herd age structure over time. Additionally, farms with HS1 and HS2 also show better productivity in terms of the number of farrowings per sow per year and fewer non-productive days, (i.e., lower values of WSI and WOI) compared to HS3 farms.

On the other hand, the census distribution of HS3 farms, with an upward concave trend and a higher percentage of sows in the latest parities (6th to 8th or older) than HS1 and HS2 farms, may be attributed to an unstructured herd [1], with lower productivity results than the other two types of herd age structure. This may be due to the higher proportion of older sows, which are less productive [4]. In addition, this structure had a lower coefficient of determination (R^2^ = 0.57) compared to HS1 and HS2 (R^2^ = 0.76 in both cases), indicating a slightly larger average difference between predicted and observed values when compared to the other two herd structure types. This difference in the coefficient of determination may be due to a higher variability of farms within this group, including farms with highly unstructured herd distribution. However, this poorer model fit could also be due to the grouping of sows from the 8th farrowing onwards, which could bias the quadratic function, especially in these HS3 farms (≥ 8th parities representing 12% of total sows), not accurately capturing the trend between the last parities. Unfortunately, this information was not available for inclusion in the models, as the BDporc dataset used groups sows from the 8th farrowing onwards.

In addition, the linear functions of the three types of herd structure, represented alongside the quadratic functions, exhibit a less precise model fit. Hence, this confirms that a quadratic regression model better fits the reality of the farms’ census structure.

These results confirm the need to consider herd age structure as a relevant factor when evaluating reproductive efficiency. They demonstrate that farms with HS2, traditionally described as the ideal or model herd [1, 3, 10–13, 32], do not achieve the best productivity results over the course of a year. Therefore, it is worth questioning whether it should be considered ideal in terms of productivity, even though it maintains a constant herd size between cycles. On the other hand, HS1 achieve the best productivity results and aligns with the herd age structure described by De Andrés et al. [2], who defined an ideal herd age structure different from that described by Carroll [10], with fewer first-parity sows and a higher percentage of sows in the most productive cycles (3rd-4th) by culling fewer sows in these early cycles and maintaining a declining herd size. However, the present study cannot confirm that HS1 is the most productive in the long term, as it has only examined the herd age structure and productivity of the farms over a year, making it very difficult to conclude the long-term effects of herd structure. Nevertheless, considering the farrowing rate of Spanish sows (2.3–2.4 per year), sows that began their productivity at the start of 2020 would have reached the end of their productive life by the end of 2022 (i.e., 2.35 farrowings per sow per year × 3 years ≈ 7 farrowings); therefore, the influence of the current herd structure would not affect productivity beyond the two following years.

In this regard, a proper herd age structure must ensure stable productivity over time, with its potential increase as a result of prolificacy, survival rate and fertility improvements. This can potentially be achieved with HS1 and HS2, provided that an appropriate replacement and culling policy is in place. For example, Mote et al. [38] suggested that producers should aim to limit sow losses to no more than 10% per parity cycle to maintain an ideal herd. However, in some farms, it may be beneficial to increase the percentage of sows in the later parities. In this context, Rodriguez-Zas et al. [18] recommended that in situations where sow costs are high, salvage or residual values are low, and revenues per piglet are also low, the optimal parity for removal should be between 6 and 10 parities. Additionally, maintaining a herd age age structure that retains mature sows allows them to reach their maximum performance [32], which depends on the management and results of each farm, and would explain why some HS3 farms can achieve good results in terms of productivity. However, it should also be considered that old sows have a higher feed consumption [26], which increases costs of production and could reduce profitability and sustainability.

On the contrary, some farms included in the HS1 group, despite having a higher number of sows in the most productive parities (from 3rd to 5t^h^) than HS2 and HS3, have a risk of reducing their productivity in the following year if current young sows (1st and 2nd parities) do not have a low culling rate to maintain the sow census in the future 3th to 5th parities. Therefore, this structure could lead to annual variations in productivity.

Considering the above, when organising a farm, it is essential to study its optimal herd age structure like any other production parameters and their targets, with the aim of maintaining a consistent replacement and culling policy over time. In any case, this study did not have information on the management techniques implemented on the farms or their health status, nor on the culling rates per parity, which represents a limitation of the study, as it would have provided relevant information to better understand the elimination patterns of different types of herd age structure, as noted by Houška [1], who evaluated how different culling rates can model the herd age structure and farm productivity efficiency. Therefore, one of the limitations of this study is the lack of data on key management practices (e.g., batch management, feeding type, gestation groups) and their interaction with culling rates. This missing data would have provided valuable insights into how management practices influence herd age structure and productivity.

Additionally, this study did not account for genetic differences among the sows, which could influence their productivity. It is assumed that the commercial genetic lines are evenly distributed across the groups of farms studied, although Iberian breed farms were excluded due to their lower prolificacy and different characteristics.

## Conclusions

The study provides valuable insights into sow distribution across parities in commercial sow-breeding farms, highlighting the importance of herd age structure in reproductive performance. The proposed classification of herd age structures, based on the coefficient “a” of the quadratic function, effectively defines the herd census structure using the curvature of the trend parabola. This approach enables a clear analysis of the association between parity distribution and farm productivity.

HS1, characterised by a downward-concave trend, shows the best productive outcomes over a year. These findings underscore the significance of herd age structure in farm management decisions and suggest that optimizing it can improve reproductive efficiency and overall farm productivity.

However, to guarantee long-term reproductive efficiency, it is necessary to evaluate how different herd structures influence productivity over consecutive years. Therefore, it is crucial to distinguish between herd structures that maximize short-term productivity and those that ensure stable medium-term performance, in order to avoid yearly fluctuations. Since this study focuses on a one-year period, future research should focus on the stability and productivity of farms according to their herd age structure over time.

## Data Availability

No datasets were generated or analysed during the current study.

## Abbreviations

BA: Piglets born alive
HS1: Herd structure type 1
HS2: Herd structure type 2
HS3: Herd structure type 3
PWSY: Number of piglets weaned per sow and year
SB: Stillborn piglets
TB: Total piglets born
W: Weaned piglets
WCI: Weaning to conception interval
WOI: Weaning-to-oestrus interval
WSI: Weaning-to-first-service interval
